# Robustness of antiadhesion between nanofibers and surfaces covered with nanoripples of varying spatial period

**DOI:** 10.3389/fevo.2023.1149051

**Published:** 2023-06-19

**Authors:** Gerda Buchberger, Marco Meyer, Cristina Plamadeala, Margret Weissbach, Günter Hesser, Werner Baumgartner, Johannes Heitz, Anna-Christin Joel

**Affiliations:** 1 Institute of Applied Physics, Johannes Kepler University Linz, Linz, Austria; 2 Institute of Biomedical Mechatronics, Johannes Kepler University Linz, Linz, Austria; 3 Institute for Biology II, RWTH Aachen University, Aachen, Germany; 4 Center for Surface and Nanoanalytics, Johannes Kepler University Linz, Linz, Austria

**Keywords:** nano spider silk, calamistrum, LIPSS, biomimetic, laser processing, PET – poly(ethylene terephthalate), SU-8, cribellate

## Abstract

Since nanofibers have a high surface-to-volume ratio, van der Waals forces render them attracted to virtually any surface. The high ratio provides significant advantages for applications in drug delivery, wound healing, tissue regeneration, and filtration. Cribellate spiders integrate thousands of nanofibers into their capture threads as an adhesive to immobilize their prey. These spiders have antiadhesive nanoripples on the calamistrum, a comb-like structure on their hindmost legs, and are thus an ideal model for investigating how nanofiber adhesion can be reduced. We found that these nanoripples had similar spacing in the cribellate species *Uloborus plumipes, Amaurobius similis*, and *Menneus superciliosus*, independent of phylogenetic relation and size. Ripple spacing on other body parts (i.e., cuticle, claws, and spinnerets), however, was less homogeneous. To investigate whether a specific distance between the ripples determines antiadhesion, we fabricated nanorippled foils by nanosecond UV laser processing. We varied the spatial periods of the nanoripples in the range ~203–613 nm. Using two different pulse numbers resulted in ripples of different heights. The antiadhesion was measured for all surfaces, showing that the effect is robust against alterations across the whole range of spatial periods tested. Motivated by these results, we fabricated irregular surface nanoripples with spacing in the range ~130–480 nm, which showed the same antiadhesive behavior. The tested surfaces may be useful in tools for handling nanofibers such as spoolers for single nanofibers, conveyor belts for producing endless nanofiber nonwoven, and cylindrical tools for fabricating tubular nanofiber nonwoven. Engineered fibers such as carbon nanotubes represent a further candidate application area.

## 1 Introduction

The uniquely high surface-to-volume ratio of nanofibers makes them ideal for various applications in drug delivery (Hu et al., 2014), wound healing (Memic et al., 2019), tissue regeneration (Jiang et al., 2015), and cell scaffolds (Wu et al., 2017). Further, they can be used as efficient filters (Desai et al., 2009; Cooper et al., 2013; Wang et al., 2016; Liu et al., 2019), in sustainable food packaging (Tien et al., 2021), and in smart textiles (Wan, 2019; Tien et al., 2021). Nanofibers are difficult to process and handle due to their inherently small size and because predominant van der Waals forces (Winterton, 1970; Israelachvili, 1974; Parsegian, 2005) make them adhesive. Similarly, nanostructures on gecko feet (Autumn et al., 2000, 2002) demonstrate the surprising strength of van der Waals forces as they enable the gecko to climb vertical surfaces. These forces are associated with the interactions between permanent and/or induced dipoles (or, more generally, multipoles).

**Figure 1:**
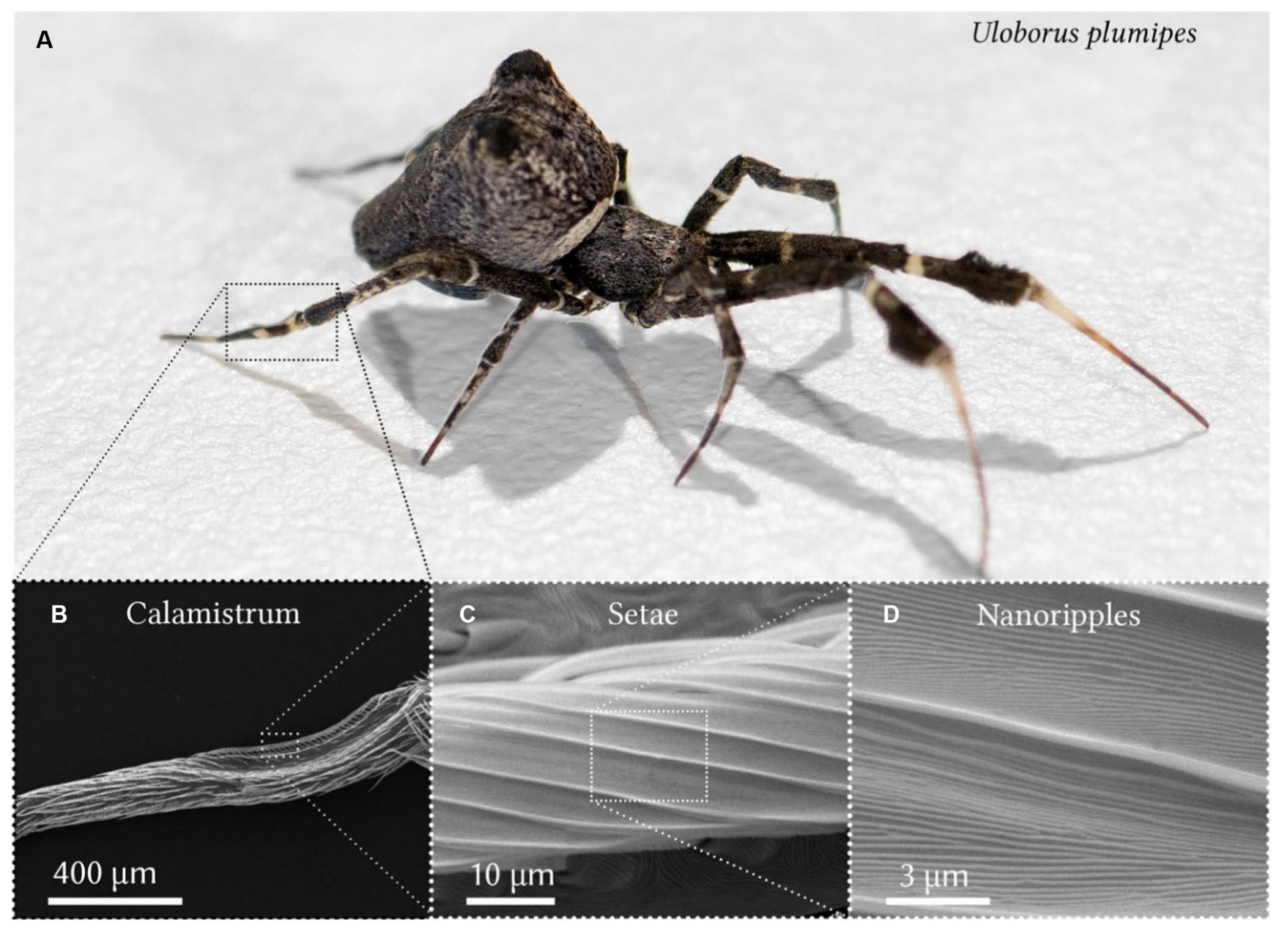
The feather-legged lace weaver *Uloborus plumipes* and its calamistrum. **(A)** The spider has a body size of about 5 mm. The calamistrum is located on the hindmost (fourth) leg. Image was kindly provided by the Bundesanstalt für Materialforschung und -prüfung (BAM). **(B)** Close-up of the calamistrum on the metatarsus of the leg. **(C)** Specialized setae of the calamistrum covered with **(D)** evenly spaced nanoripples. **(B-D)** Scanning electron microscopy images of gold-sputtered specimens.

With cribellate spiders, nature offers inspiration for overcoming nanofiber adhesion (Joel et al., 2015, 2020; Meyer et al., 2021; Figure 1), as they are capable of producing, processing, and handling nanofibers (Hawthorn and Opell, 2002; Joel et al., 2015, 2016; Bott et al., 2017; Grannemann et al., 2019). Cribellate spiders, a paraphyletic group within the web-building spiders (Araneae), incorporate thousands of ~15–30 nm thick nanofibers into their capture threads to give them adhesive properties (Friedrich and Langer, 1969; Peters, 1992; Opell and Schwend, 2007; Joel et al., 2015, 2023; Kronenberger and Vollrath, 2015; Bott et al., 2017; Joel and Baumgartner, 2017). They form them into wooly puffs that surround thicker axial fibers, e.g., in Uloboridae (Opell, 1979; Peters, 1984; Joel et al., 2015). To brush the nanofibers into this characteristic voluminous structure during extraction from spigots, the spiders use a comb-like structure: the calamistrum (Montgomery, 1909; Joel et al., 2015, 2016, 2020) on the metatarsus of each of their hindmost (fourth) legs (Figure 1B). Despite frequent contact, nanofibers do not adhere to the calamistrum (Joel et al., 2020). Different species (e.g., Uloboridae and Deinopidae) show morphological differences in their calamistra (Joel et al., 2016).

Joel et al. (2020) demonstrated that, in the cribellate feather-legged lace weaver *Uloborus plumipes* (*U. plumipes*), these antiadhesive properties are due to a rippled nanotopography found on the setae of the calamistrum (Figure 1D). Such ripples can be found in the majority of species with similar dimensions of about 200–300 nm in both periodicity and height (Ramírez, 2014; Joel et al., 2016; Heiss et al., 2018; Lifka et al., 2022). It has been demonstrated that similarly sized nanoripples reduce the adhesion between nanofibers of *U. plumipes* and biomimetic nanorippled surfaces (Joel et al., 2020; Meyer et al., 2021).

For the self-organized formation of nanoripples on these artificial surfaces, laser-induced periodic surface structures (LIPSS; Bäuerle, 2011; Bonse et al., 2017; Stratakis et al., 2020) on poly(ethylene terephthalate) (PET) were used. In combination with a gold-coating, biomimetic LIPSS on PET foils led to a total reduction in nanofiber adhesion of more than 70% compared to the noncoated unstructured reference foils (Joel et al., 2020). This is due to minimization of the contact area between the nanofibers and the surface, which in turn reduces the van der Waals forces (Joel et al., 2020). The stability of antiadhesion between biomimetic foil with LIPSS and nanofibers was tested over a temperature range of 10–40°C and a relative humidity range of 10–90% (Meyer et al., 2021). Antiadhesion remained robust under moderate ambient conditions, decreasing only at a relative humidity of 70% and at temperatures ≥30°C.

To produce LIPSS (i.e., self-organized surface nanoripples on solids or liquids after laser irradiation; Bäuerle, 2011; Bonse et al., 2017), the laser light must be polarized and the processing parameters within a certain range. According to the simplest theory, LIPSS arise from the interference pattern between the incident laser beam and the light scattered by a surface with nanoscale roughness. Fabrication of LIPSS on commonly used technical polymers such as PET (La Mantia et al., 2013) or epoxy-based photoresist SU-8 (Abgrall et al., 2007; Ceyssens and Puers, 2012) requires thousands of nanosecond (ns) laser pulses with a fluence well below the single-pulse ablation threshold hitting the exact same spot. In the case of PET and SU-8, the LIPSS produced are oriented in parallel to the direction of the linear polarization. The spatial period *Λ* of LIPSS fabricated using s-polarization depends on the angle of incidence *θ* of the laser light and is given by Λ = *λ* / (*n*_eff_ − sin *θ*). Here, *λ* is the wavelength of the laser light and *n*_eff_ is the effective refractive index which lies between the refractive indices of air and of the polymer used. LIPSS on SU-8 have previously been used for surface-enhanced Raman spectroscopy platforms (Kalachyova et al., 2017; Guselnikova et al., 2019; Erzina et al., 2020) and LIPSS on PET for activation of human cells (Barb et al., 2014) and formation of gold nanowires (Siegel et al., 2010; Barb et al., 2014; Steinhauser et al., 2018; Heitz et al., 2020). Compared to pristine PET, LIPSS on PET reduce the adhesion of *Escherichia coli* bacteria by ~91% (Richter et al., 2021); here, pili – nanofiber-like appendages of bacteria – and the spatial period of the nanoripples have been shown to play a crucial role in adhesion.

Although structure and composition of cribellate capture threads vary widely between species (Friedrich and Langer, 1969; Eberhard and Pereira, 1993; Opell, 2002; Grannemann et al., 2019), a previous study found that the calamistrum nanoripple dimensions (i.e., nanoripple height and spacing) are very similar even for the distantly related species *Hickmania troglodytes, Uloborus plumipes, Jamberoo johnnoblei*, and *Amaurobius similis* (Lifka et al., 2022). We wondered why the calamistrum is so homogeneously structured for these species and hypothesized that its specific dimensions might be related to its antiadhesive properties. The biomimetic antiadhesive foils presented by Joel et al. (2020) and Meyer et al. (2021) should then be least adhesive at a similar spatial period of ~203 nm.

Investigating the species *Uloborus plumipes, Amaurobius similis*, and *Menneus superciliosus*, we determined the distances between the nanoripples of the calamistrum and of other spider body parts, including the claws, cuticle and spinnerets. We found that the spatial periods of the nanoripples on the calamistrum matched closely, while those on the claws, cuticle, and spinnerets varied widely. The function of these varying ripples is unknown, but all of these organs are likely to come into contact with nanofibers. We further investigated whether the antiadhesive properties of nanoripples rely on precise structural dimensions or whether, as suggested by the dimensions of the nanoripples on the other body parts, a wider range of spatial periods is functional. In a biomimetic approach, we fabricated LIPSS on PET foils and SU-8 films using a KrF laser to vary ripple spacing and height in the ranges ~203–613 nm and ~63–161 nm, respectively. We chose PET and SU-8 because we needed flexible, bendable foils for the antiadhesion measurements. To assess the antiadhesive properties, we used natural cribellate capture threads, since nanofibers with diameters as small as 15–30 nm have to date not been manufactured at an industrial scale. The tested surfaces may find application in tools for working with small-diameter nanofibers, for example, in spoolers (for single nanofibers) and in conveyor belts and cylindrical tools (for producing endless and tubular nanofiber nonwoven, respectively). Furthermore, they may be applicable to alternative fibers such as carbon nanotubes.

## 2 Materials and methods

### 2.1 Study animals

Adult *Uloborus plumipes* (Lucas, 1846) specimens were caught in garden centers in Aachen, Germany. The spiders lived separately in boxes with a size of 11 cm × 11 cm × 6.5 cm and with roughened surfaces. These boxes served as their cages. Spiders had similar size and could build their webs under laboratory conditions, i.e., at room temperature (approx. 20°C), at relative room humidity (approx. 30%), and experienced a diurnal rhythm of a 12 h light period followed by a 12 h dark period. Their diet consisted of *Drosophila melanogaster* specimens and occasionally small crickets or cowpea weevils, which we fed them weekly. Soaked cotton balls served as water supply biweekly. Capture threads of the webs were collected for measuring the antiadhesive properties of the biomimetic samples.

Adult *Amaurobius similis* (Blackwall, 1861) specimens were caught near Gut Melaten in Aachen, Germany. One adult and further juvenile *Menneus superciliosus* (Thorell, 1881) specimens were caught in the Blue Mountains, in the forest between Megalong Road and Pulpit Hill Creek close to Blackheath, Australia. Both species were sacrificed in 70% ethanol and later dried for SEM analysis (Section 2.4.1).

All species used in the experiments are neither endangered nor protected. No special permits were required. All applicable, international, national, and institutional guidelines for the care and use of animals were followed.

### 2.2 Fabrication of unstructured and structured test surfaces

#### 2.2.1 Manufacturing of SU-8 thin films on PET foils

SU-8 is a negative photoresist and is widely applied as a structural material for labs-on-chips, microelectromechanical systems, and microelectronics (Abgrall et al., 2007; Ceyssens and Puers, 2012). SU-8 has made the fabrication of high-aspect-ratio structures accessible to labs with no high-end facilities because a standard UV lithography process is applicable. In the future, this process could be even combined with LIPSS. In contrast to acrylic resin photoresists, epoxy-based SU-8 thin films are bendable.

SU-8 thin layers were deposited onto PET foils using spin-coating. First, the PET foils (Goodfellow Ltd., Bad Nauheim, Germany) were cleaned of all impurities by consecutively immersing them in acetone, ethanol, and distilled water and putting them into an ultrasonic bath (Bandelin Sonorex RK 255H, Berlin, Germany) for 10 min for each solvent. After sonication, the samples were thoroughly dried. In the next step, 500 ml of SU-8 2005 (Kayaku Advanced Materials, Inc., Westborough, USA) was deposited onto the clean PET foil and spin-coated for 5 s at 500 rotations per minute (rpm) and for 30 s at 2000 rpm (spin-coater from Micro Tech Mfg. Inc., Worcester, USA). The samples were then pre-baked (in an in-house made oven) at 65°C for 10 min for the solvent to be evaporated and the film densified. After that, the samples were taken out from the oven, and allowed to cool down at room temperature. In the last step, the SU-8 layer was cross-linked by exposing it to an ultraviolet (UV) lamp (UV-Belichtungsgerät, isel, proMA Technology GmbH; lamps: PHILIPS TLD 15 W/05, 300 nm – 460 nm with a peak at 365 nm) for 2 min. Typical layer thicknesses obtained by this procedure are in the range of 5 μm.

#### 2.2.2 Laser processing of PET and SU-8 for formation of surface nanoripples

The biomimetic nanoripples on PET described by Joel et al. (2020) and Meyer et al. (2021) as well as the LIPSS applied by Siegel et al. (2010), Barb et al. (2014), Steinhauser et al. (2018), Heitz et al. (2020), and Richter et al. (2021) were fabricated by a ns KrF excimer laser emitting UV light with a wavelength of 248 nm. The very same laser type was also used in this study.

**Figure 2:**
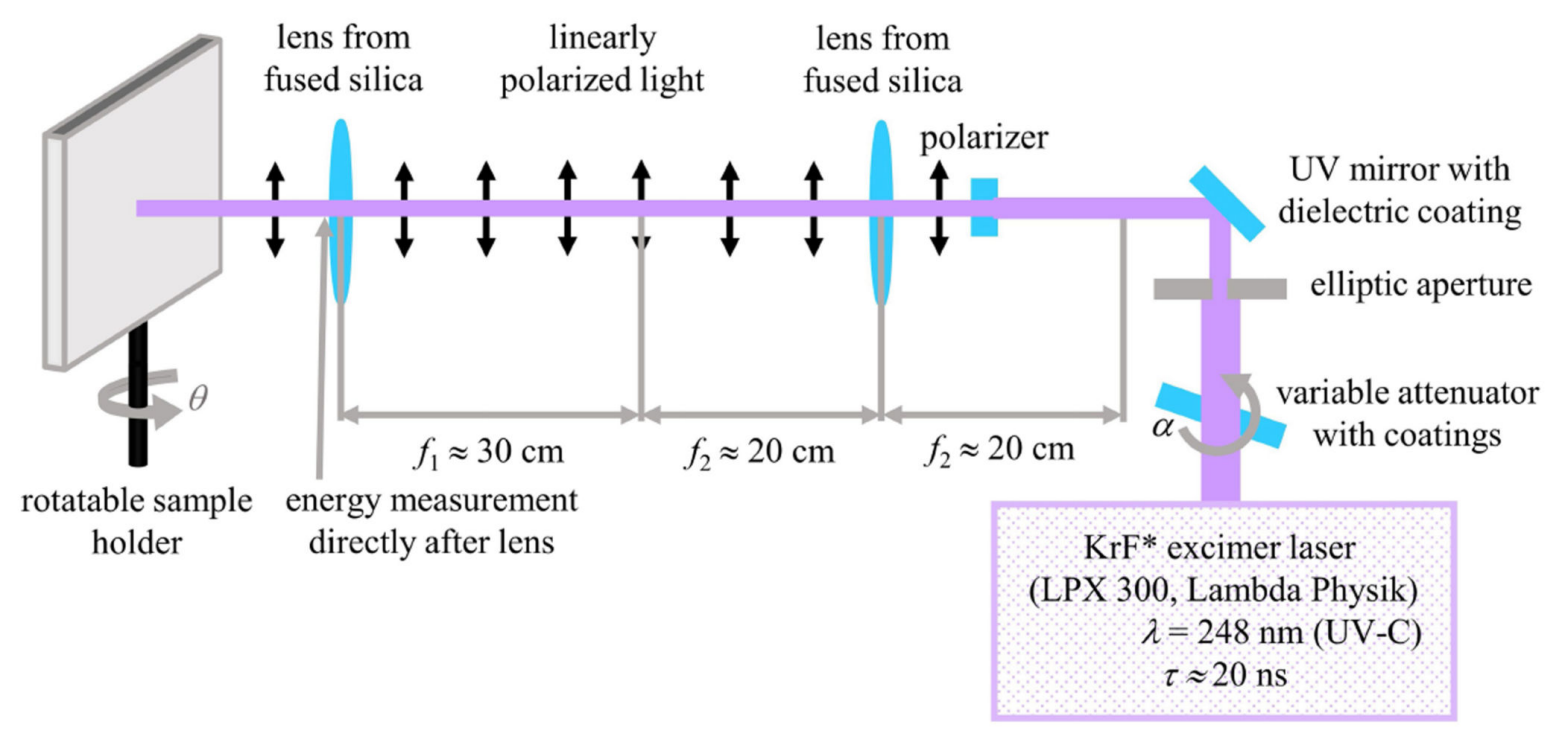
Experimental setup for the fabrication of nanoripples on PET and SU-8 using a ns UV laser. Adapted from Meyer et al. (2021) and Richter et al. (2021).

Flat, 50 μm thick, biaxially stretched PET foils (Goodfellow Ltd., Bad Nauheim, Germany) were used as the first base material for nanorippled surface production; the second base was a 5 μm thick SU-8 layer on the very same PET foils (see Section 2.2.1 for details on the fabrication). The base materials were treated with ns UV-C laser light using a KrF excimer laser (LPX 300, Lambda Physik, Göttingen, Germany) with a wavelength of *λ* = 248 nm and a pulse duration *τ* of about 20 ns (Figure 2); the pulse repetition rate was set to *ν* = 10 Hz. For the formation of regular nanoripples, the laser was operated in the constant voltage mode using 22 kV and the laser light was linearly polarized either using an *α*-BBO polarizer (Melles Griot, Carlsbad, CA, USA) in the case of the PET surface or by means of another polarizer (Thorlabs GmbH, Bergkirchen, Germany) in the case of the SU-8 surface. Two lenses from fused silica were mounted to form a telescope that imaged the output of the polarizer onto the samples. The pulse energy was measured by a pyroelectric joulemeter (model ED-500, Gentec from Soliton Laser- und Messtechnik GmbH, Gilching, Germany) directly after the second lens before a rotatable sample holder. With this sample holder, the angle of incidence *θ* was increased in steps of 10° from 0° to 60°. For laser processing of PET, *N* = 6,000 pulses were used with an average fluence *Φ* of approximately 5.7 to 6.2 mJ/cm^2^. For LIPSS formation on SU-8, 3,500 pulses were applied with an average fluence *Φ* of approximately 6.2–7.2 mJ/cm^2^. In Richter et al. (2021) the effective refractive index for PET was calculated from seven measurement points to be 1.235 ± 0.053 (± 4.3%) at the applied average fluences of approximately 5.7–6.2 mJ/cm^2^.

The pulse energy was increased with the angle of incidence because the area of the laser spot *A_θ_* grew according to the formula *A_θ_* = *A*_0_°/cos*θ*, where *A*_0_° is the smallest processed area, where the laser hits the sample vertically with an angle of incidence *θ* = 0°. The size of the laser-processed area was dependent on the angle of incidence and was typically between one to three square centimeters. For the formation of the irregular nanoripples, a second beamline without lenses and a polarizer was established. A fixed sample holder was mounted and aligned vertically to the laser beam. The laser was operated at 18 kV so that the pulse energies were reduced and the fluence could be kept constant despite a processed area smaller in size. The energy was measured directly before the fixed sample holder with the same joulemeter as described before. Adhesive tape was used to glue the samples on the holders. The pulse energy was adjusted using a high-power variable attenuator with a dielectric coating (magnetron sputtered and with an antireflection coating, Laseroptik GmbH, Garbsen, Germany) which was mounted onto an electronically controlled, rotatable stepper motor. For each parameter combination, at least six samples were fabricated which were then used for further measurements.

### 2.3 Focused ion beam cuts of artificial surfaces

Focused ion beam (FIB) cuts were performed instead of AFM measurements because in previous studies significant convolution of the surface morphology with the geometry of the AFM tip was observed (Meyer et al., 2021), wherefore the respective ripple heights turned out to be most probably not fully correct (Richter et al., 2021). A gold layer of approximately 12 nm was deposited *via* sputter coating (AE1230, EMScope, Ashford, UK; 4 min at a deposition current of 20 mA at a power of 14.3 W), to prevent charging and to enhance the contrast between the surface of the sample and the protective deposit on top of the gold layer. A dot from a black permanent marker pen was used (Lumocolor, Staedtler Mars GmbH & Co. KG, Nürnberg, Germany) as a protective deposit directly at the position of the planned FIB cut. At the rim of this dot, where the protective layer is thinner than in the middle, rectangles or trapezoids with approximate sizes between 10 and 15 μm were cut into the material by using a milling current of 200–500 pA and an acceleration voltage of 30 kV. A 1540XB-Crossbeam (Zeiss, Oberkochen, Germany) was applied which combines a GEMINI^®^ field-emission scanning electron microscope for imaging (FE-SEM) and a FIB device for cutting. The ion source was a Ga^+^ − filament.

Focused ion beam cuts were evaluated using the Fiji distribution of the free software ImageJ2 (versions 1.52 s, National Institutes of Health, Bethesda, MD, USA). The tops and valleys of the nanoripples were marked with the “Multi-point” tool; then the positions of the tops and the valleys were extracted *via* the “Measure” tool. The heights of the ripples were calculated from the differences between the tops and the valleys by using Excel (Microsoft Office 16, Microsoft Cooperation, Redmond, WA, USA). For each height value, the distances between one top and the valleys to its left and its right were averaged. The scale bars of the SEM images of the FIB cuts were used to transform all values from pixels into nanometers. For each exemplary position, 20 ripples were measured to compute the mean values and standard deviations. In the images of the FIB cuts the tilt correction is switched on which leads to a correct depiction of the ripple heights in the vertical direction and an elongation in the horizontal direction, wherefore horizontal distances and spatial periods are not displayed correctly.

### 2.4 Microscopy

#### 2.4.1 SEM of the body of spiders

Three specimens of the species *U. plumipes* were sacrificed by freezing and air-dried, whereas those of the species *A. similis* and *M. superciliosus* were stored in ethanol (70%, AppliChem GmbH, Darmstadt, Germany) and then dried *via* a drying series: increasing concentrations of ethanol, namely from 80, 90, to 100%, followed by increasing concentrations of ethanol: hexamethyldisilazane (HDMS), namely from 3:1, 1:1, 1:3, to 0:1 (HDMS ≥98% Carl Roth GmbH & Co., Karlsruhe, Germany). Afterward, metatarsi and opisthosoma were detached from the spiders and transferred to SEM-stubs, ensuring that the tarsal claws, the calamistra, and the spinnerets were freely visible. All specimens were examined with the SEM (SEM 525 M; Philips AG, Amsterdam, Netherlands) after sputter-coating them with an approx. 10 nm thick gold layer (Hummer Technics Inc., Alexandria, VA, USA; applying a current of 7.5 mA at a voltage of 1,000 V for 5 min). The structures’ spatial periods were measured using ImageJ2 (version 1.53 t, National Institutes of Health, Bethesda, MD, USA). Fifteen values per body part were measured.

#### 2.4.2 SEM of PET and SU-8 surfaces

A gold layer with a thickness of 8 to 10 nm was deposited by means of a sputter coater (AE1230, EMScope, Ashford, UK; 3 min at a deposition current of 20 mA at a power of 14.3 W) before SEM imaging (model REM 1540XB-Crossbeam, Zeiss, Oberkochen, Germany).

#### 2.4.3 Computation of spatial periods *Λ* from SEM images

The spatial periods *Λ* of the regular LIPSS at PET and SU-8 surfaces were calculated as described in Richter et al. (2021) by using the free software Gwyddion (version 2.55, Czech Metrology Institute, Brno, Czech Republic). First, the two-dimensional fast Fourier transforms (2D FFT) of SEM micrographs were computed at one position on the sample (magnification of 15.14 k-times and size of 14.83 × 19.77 μm^2^) by using the default conditions (output type “modulus,” window type “Hann” and “Subtract mean value beforehand”). Depending on their spatial periods between 30 and 100 ripples were evaluated. Then the profiles were extracted along the lines through the resulting peaks to which Lorentzian functions L(*k*) = *y*_0_ + *a*/[*b*^2^ + (*k* – *k*_0_)^2^] were fitted; here, *k*_0_ represents the position of the peak. The distances Δ*k* = *k*_0,right_ – *k*_0,left_ between the peaks which were situated, respectively, at the left and at the right sides of the central peak at the positions *k*_0,left_ and *k*_0,right_ were calculated. The spatial periods were computed directly from these distances by applying the formula *Λ* = 2/Δ*k* = 2/(*k*_0,right_ – *k*_0,left_). The Gaussian law of propagation of uncertainty was used to determine the errors of *Λ* from the errors of the peak positions given by Gwyddion’s Lorentzian fit function; these values were then used to estimate the relevant digits of the given spatial periods. The distances of the irregular ripples fabricated at an angle of *θ* = 0° without a polarizer had to be measured directly from profiles in SEM images as no distinct peaks were visible in the Fourier transforms. Profiles from SEM images were extracted along lines vertically to the ripples using Gwyddion and then the peak-to-peak distances were measured directly in the resulting graphs with the very same software.

### 2.5 Antiadhesion measurements

**Figure 3:**
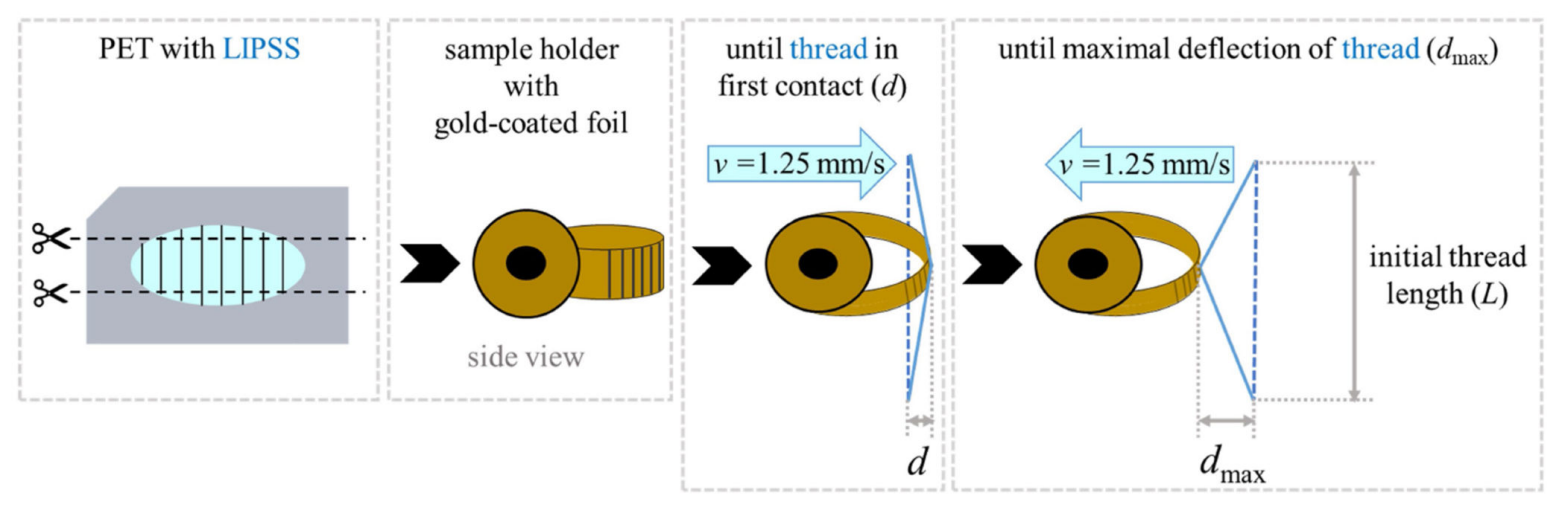
Principle of adhesion measurement. **(A)** Structured and unstructured foils were cut into strips. **(B)** Then they were bent into a loop form and gold-coated in this configuration. **(C)** For measurements, a thread with a defined length was moved until it was deflected through contact with the foil by ~500 μm (*d*). **(D)** After 3 s the sample was withdrawn and the maximal deflection (*d*_max_) was measured when the thread detached.

Biomimetic foils with surface nanoripples were tested to assess their antiadhesive properties; unstructured foils without any laser treatment served as control samples. Before each experiment, fresh threads were sampled from spider webs with an *L* = 7 mm wide sample holder (Figure 3). Every thread was only used once. To account for the structural variability of spider silk (i.e., puff sizes, number, and orientation of nanofibers, and silk composition) between different individuals, threads from the same four to six spiders for each type of foil were used. The exact same holder was used for all threads, which ensured equal lengths. Double-sided adhesive tape allowed us to fix the threads tightly onto the holder. Unsuitable threads, i.e., threads with visibly impaired macrostructure, were identified by light microscopy before and after the experiments and were discarded.

For testing an antiadhesive effect on the threads, specially mounted foil strips were used (Figure 3). As described also by Joel et al. (2020) and Meyer et al. (2021) the foil strips were clamped in a loop form to avoid the formation of edges to which fibers could entangle. These strips were approx. 3 cm long and approx. 1 cm wide. They were used for several measurements, moving the threads to a new position on the foil before each experiment. All foils, the structured as well as the unstructured ones, were sputter-coated in the loop form with a 10 nm thick layer of gold (same parameters and device as in Section 2.4.1). The gold coating had three purposes: (1) To reduce electrostatic charging of the polymer foils. (2) To adapt the surface chemistry of the two base materials PET and SU-8 so that all matched. (3) To attain the same surface chemistry of non-processed, i.e., unstructured, and laser-processed, i.e., nanorippled, materials because KrF laser treatment is known to change the chemical composition of PET making it more hydrophilic or hydrophobic depending on the applied number of laser pulses and fluence (Watanabe and Takata, 1993; Wong et al., 2001; Mirzadeh et al., 2011; Richter et al., 2021). The foils were additionally grounded to decrease electrostatic charging.

The holder with the thread was mounted on a motorized micromanipulator (MT30-50; Standa Ltd., Vilnius, Lithuania) and approached the foils with a velocity of *v* = 1.25 mm/s. The thread deformed slightly to a distance *d* of ~500 μm from the position of the non-extended thread, indicating contact with the foil. It stayed for 3 s in this configuration, whereupon it was slowly withdrawn from the surface with a constant speed of *v* = 1.25 mm/s until the thread detached with a maximum deflection of *d*_max_ compared to the non-extended thread. When the influence of electrostatic attraction was suspected, the air was ionized *via* an ion gun (Milty Zerostat 3; Armour Home Electronics Ltd., Bishop’s Stortford, UK).

For all experiments, the orientation of the nanoripples was chosen to be perpendicular to the thread. Videos of the deflections were recorded with 60 fps at 10x to 20x magnification (VW-9000C; Keyence Corporation, Osaka, Japan). Twenty positions on each nanorippled (biomimetic) surface manufactured under different processing parameters were tested as well as 20 positions on unstructured (control) surfaces. To avoid systematic influences, the experiments were shuffled and carried out on different days. As the ambient climate was shown to influence the antiadhesion between nanofibers and biomimetic foils (Meyer et al., 2021), the relative humidity (from 27 to 57%) and the temperature (from 24.1 to 26.6°C) were tracked during the measurements (Reptiland; Trixie Heimtierbedarf GMBH & Co.KG, Tarp, Germany). The temperatures were measured near the threads as the light sources heated up the air around the threads up considerably compared to room temperature. Data were excluded when the entanglement of fibers or significant electrostatic attraction before or during antiadhesion experiments was observed. To compensate for excluded data, additional measurements were performed to keep the total number of successful experiments constant. Adhesion was determined by measuring the maximum deflection perpendicular to the initial thread position using the Keyence VW-9000 motion analyzer software (version 1.4.0.0, Keyence Corporation, Osaka, Japan).

### 2.6 Statistical analysis

Matlab R2015a (The MathWorks, Inc., Natick, MA, USA) was used for statistical analysis. A distribution test was performed, namely a one-sample Kolmogorov–Smirnov test to determine, if the measurement data came from a standard normal distribution at the 5% significance level; to this end, the data were centered and normalized using the mean value and the standard deviation. Then another distribution test was performed, namely a Lilliefors test to assess the same thing with an alternative test method (usage of default *p* < 0.05 significance level). An analysis of variance (ANOVA) techniques test, namely a one-way ANOVA, was used to determine whether the set of mean values from the adhesion or height measurements from different sample types were equal or not. This result did not provide information on which sample types were different regarding their mean values. Multiple pairwise comparisons of the mean values were performed using Tukey’s honestly significant difference criterion (usage of default *p* < 0.05 significance level and Tukey–Kramer type of critical value). Multiple pairwise comparisons were not needed for the analysis of the difference in spatial periods of the two sample types only. Data were compared individually and the respective *p*-values were assessed using a Wilcoxon rank sum test. The latter tests the null hypothesis that the data are samples from continuous distributions with equal medians, against the alternative that they are not. Significance was assumed if *p* < 0.05.

## 3 Results

### 3.1 Characterization of the surfaces covered with nanoripples

#### 3.1.1 Biomimetic surfaces from PET and SU-8

**Figure 4:**
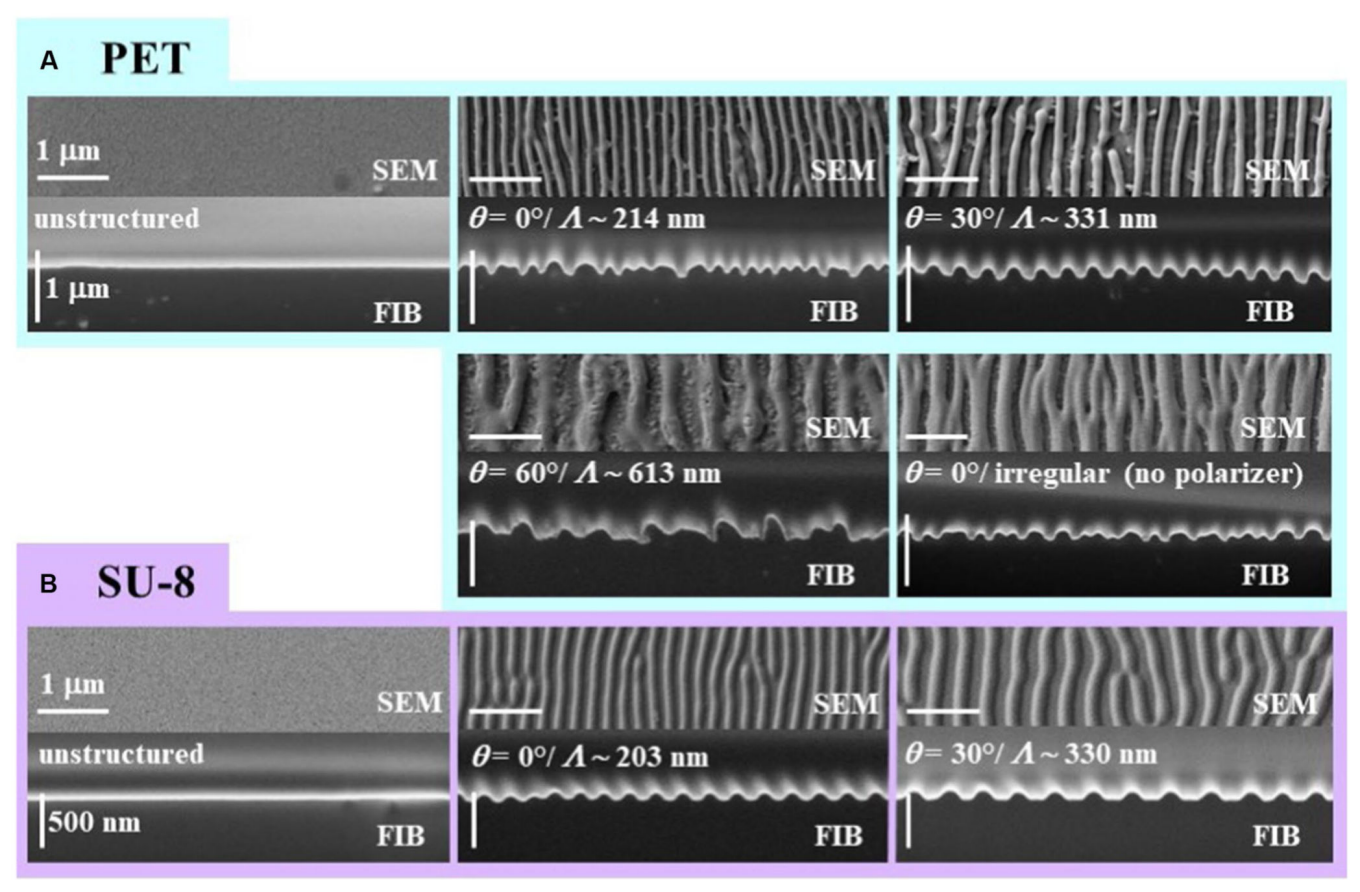
SEM images and FIB cuts of biomimetic nanorippled surfaces from **(A)** PET and **(B)** SU-8. Each framed inset shows a sample type with the SEM image at the top and the FIB cut at the bottom; turquoise frames are used for PET and purple ones for SU-8. At the very left the unstructured pristine surfaces are shown which serve as reference samples in the antiadhesion measurements. The spatial periods *Λ* of the ripples increase with the angle of incidence *θ* of the linearly polarized laser beam. In case the polarizer is taken out from the beam path the ripples will become irregular (bottom right inset of **A**). please note that in the images of the FIB cuts the tilt correction is switched on which leads to a correct depiction of the ripple heights in the vertical direction and an elongation in the horizontal direction.

We analyzed SEM images and FIB cuts of the biomimetic nanorippled PET and SU-8 surfaces to assess their morphology (Figure 4). We computed spatial periods from SEM images (images in the top rows of Figure 4) using 2D FFT (Section 2.4.3) at one exemplary sample position on the laser-processed area (Table 1). The spatial periods *Λ* of the fabricated ripples range from ~203 nm for *θ* = 0° on SU-8 to ~613 nm for *θ* = 60° on PET. The absolute standard deviations are very small, ranging from tenths of nanometers to several nanometers; the respective relative standard deviations are typically several tenths of a percent. The distances of the irregular ripples fabricated with an angle of *θ* = 0° without a polarizer had to be measured directly from profiles in SEM images as there no distinct peaks were visible in the Fourier transforms; they vary in a wide range between 130 nm and 480 nm. Their mean value ± standard deviation is 274 nm ± 91 nm (± 33%; *n* = 30) and their median value is 250 nm. Additionally, for the two sample types fabricated with *θ* = 30° the spatial periods were acquired at five positions on one sample to estimate the variances all over the square centimeter-sized processed area (Table 2). We find the nanoripples on PET to be more homogeneous showing a spatial period of 327 nm ± 6 nm (± 2%; *n* = 5) compared to the nanoripples on SU-8 with a spatial period of 318 nm ± 12 nm (± 4%; *n* = 5). In both cases, the relative deviations of 2 and 4% are very small and do not differ significantly (*p* = 0.22 from the Wilcoxon rank sum test and *p* = 0.16 from one-way ANOVA; with significance assumed at *p* < 0.05). The data can be treated as normally distributed (according to the Kolmogorov–Smirnov test *p* = 0.97 for PET and *p* = 0.085 for SU-8 and significance assumed at *p* < 0.05; for Lilliefors test *p* is greater as the largest tabulated value).

We measured the heights of the ripples from FIB cuts (Section 2.3 and images in bottom rows of Figure 4) at two exemplary sample positions on the laser-processed area (Table 3); at each position, 20 ripples were evaluated. According to the Kolmogorov– Smirnov test, all data can be assumed to be normally distributed except for the heights measured at the first position of the SU-8 sample fabricated with *θ* = 0°. According to the Lilliefors test, even more height data is not normally distributed: the heights of LIPSS on PET processed with *θ* = 30°, the heights of irregular ripples on PET measured at the second position as well the heights of the SU-8 ripples with *θ* = 0° assessed at the first position. We find by ANOVA and multiple pairwise comparisons (Section 2.6), that the heights of the ripples on SU-8 with mean values between ~63 nm and ~75 nm are significantly shorter than the ones on PET with mean values between ~97 nm and ~161 nm except for the second position on PET fabricated with *θ* = 0° (mean height of 63 nm); the measurement values of all investigated SU-8 samples do not differ significantly from each other. The significantly highest ripples can be found at the first measurement position of the sample fabricated with *θ* = 30° and at the second measurement position of the sample fabricated with *θ* = 60°, respectively, being 161 nm ± 21 nm (±13%) nm and 150 nm ± 48 nm (±32%). The SU-8 samples show fewer relative variations in the heights (between ~7% and ~18%) than the PET samples (between ~13% and ~47%).

The most homogeneous PET sample is the one fabricated with *θ* = 30° with relative standard deviations of 13% and 16% at the two measurement positions. Both the increased mean heights as well as the larger inhomogeneity of the PET ripples are thought to be attributed to the fact that more pulses are used in the fabrication of the respective samples (6,000 pulses compared to 3,500 pulses).

#### 3.1.2 Body of the spiders

**Figure 5:**
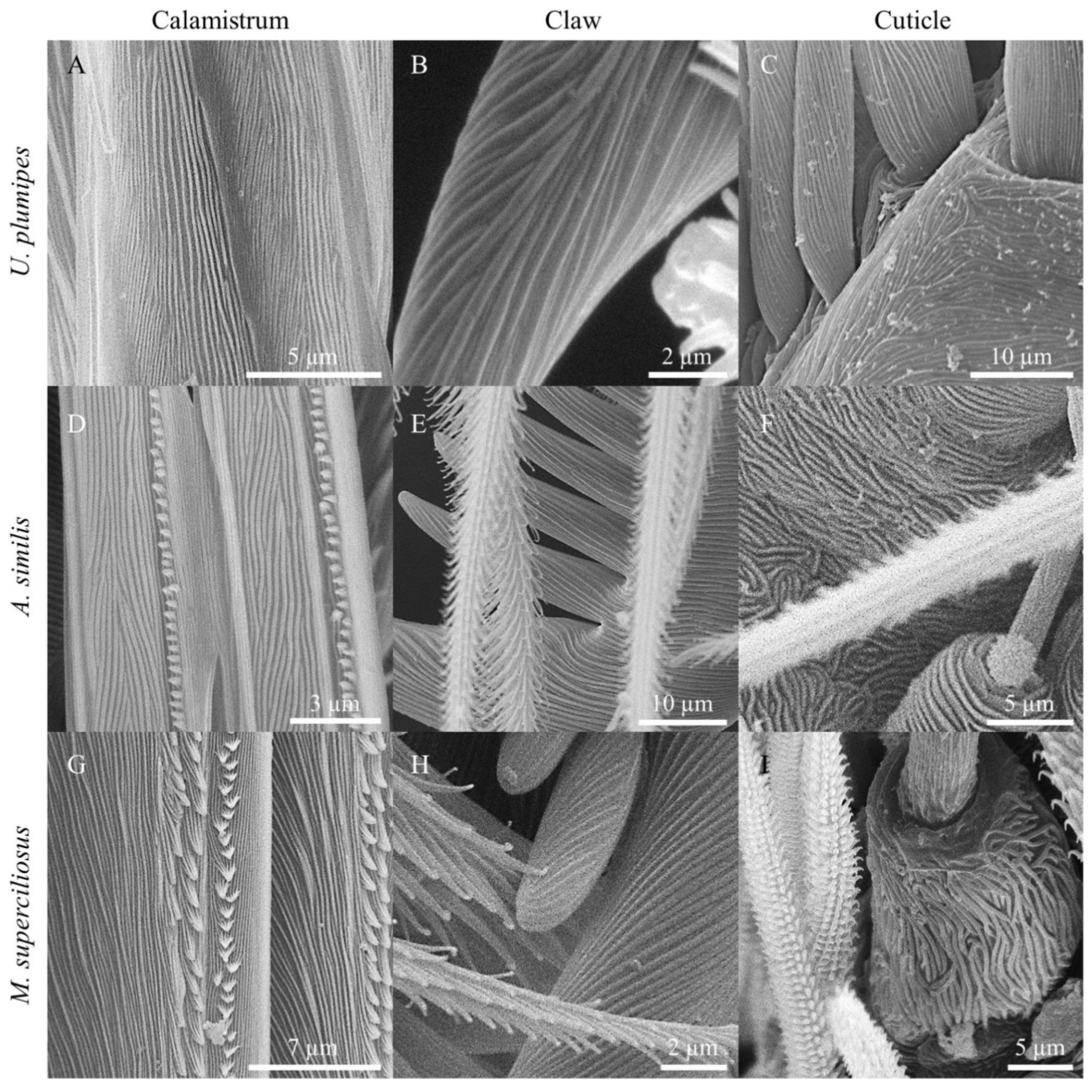
Nanoripples on different body parts of the cribellate spider species *Uloborus plumipes, Amaurobius similis*, and *Menneus superciliosus*. **(A)** Calamistrum, **(B)** claw, and **(C)** cuticle of *U. plumipes*. **(D)** Calamistrum, **(E)** claw, and **(F)** cuticle of *A. similis*. **(G)** Calamistrum, **(H)** claw, and **(I)** cuticle of *M. superciliosus*. The spatial periods of the biological nanoripples corresponded very closely to those of the LIPSS and also resembled them in their morphological expression.

Nanoripples with comparable spatial periods as on our artificial samples were found on the calamistrum, tarsal claws, spinnerets, and spigots as well as on the cuticle of the metatarsi in all three species (Table 4; Figure 5). The spatial periods of the calamistra in all three species matched the data presented by Lifka et al. (2022) and were close to the periodicity of the LIPSS produced at an angle of incidence of 0°. Nanoripples on the spinnerets, on the other hand, were comparable to LIPSS manufactured at a 60° angle of incidence. Structures on the cuticle and claws differed more between species but were within the periodicity range of the LIPSS produced except for the ones on the cuticle of *U. plumipes* which are larger.

### 3.2 Antiadhesion measurements

**Figure 6:**
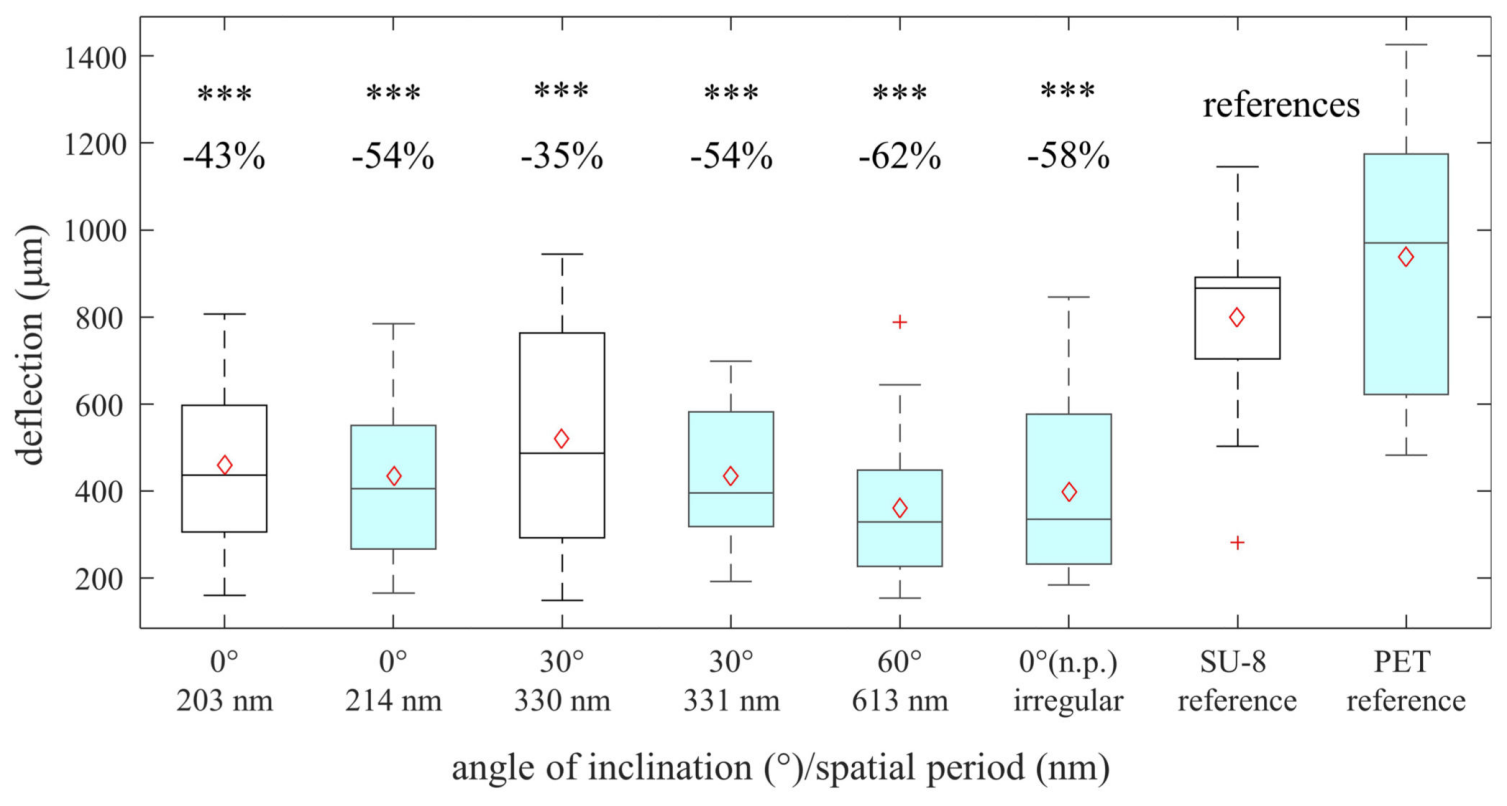
Indirect measurement of antiadhesion of gold-covered nanorippled SU-8 (unfilled black boxes) and PET surfaces (filled turquoise boxes). Shown are box-whisker plots of maximal deflections of the threads before detaching from the surface (*n*=20). In the area above, the difference in the deflections between the references and structured material (in %) is listed as well as the statistical relevance. Significant differences between LIPSS and the references were determined by a Wilcoxon rank sum test. “***” stands for *p*<10^−3^. The box-whisker plots present the 25^th^ and 75^th^ percentiles within the box along with mean (open red diamonds) and median (continuous line) values and statistical outliers (red crosses). The whiskers extend to the most extreme data points not considering the outliers.

Figure 6 shows box-whisker plots of the measured maximal deflections of the threads before detaching from the surface depending on the sample types, i.e., the applied base materials, angles of incidence *θ*, and resulting spatial periods *Λ*. The measured mean values ± standard deviations are listed in Table 5 along with the reduction in mean adhesion compared to the respective reference samples. All measurement data can be treated as normally distributed; the respective *p*-values are given in Table 6 (with significance assumed at *p* < 0.05). The result of the one-way ANOVA states that the sample type mean values are not equal with a *p*-value of 8.06 ⋅ 10^−19^. Multiple pairwise comparisons indicate that the ripples at SU-8 and PET reduce the mean adhesion of the nanofibers significantly compared to the reference samples of both materials (Table 7; significance assumed at *p* < 0.05). The reference values are, respectively, 801 μm ± 194 μm for SU-8 and 937 μm ± 299 μm for PET. Indeed, the mean maximal deflections are lowered by ~ − 35% to ~ − 62%. The differences in the median values are significant as well (Table 5 with *p*-values from Wilcoxon rank sum tests; significance assumed at *p* < 0.05). Here, ripples on PET reduced the mean deflections by ~ − 11% to ~ − 23% better than ripples on SU-8; this not significant difference might be attributed to the fact that most measured ripples on PET are significantly higher than the ones on SU-8 (Section 3.1.1). We find that according to the statistical tests, the antiadhesive performance of all laser-processed sample types does not differ significantly from each other and that both unstructured reference samples feature similar mean displacements. Interestingly enough, also the irregular ripples with peak-to-peak distances between 130 nm and 480 nm, which were fabricated without a polarizer, reduced the adhesion by about ~ −58%.

## 4 Discussion

For technical reproduction and applications, it is key to know how the system will react to changes in the ripple dimensions, that is, to variations in ripple height and spacing, and to less homogeneous ripple distribution. A possible effect of such changes was shown by Richter et al. (2021), where an increase in the spatial period of LIPSS to ~613 nm led to a complete failure to repel bacteria, though repellency had been achieved for LIPSS with a periodicity of ~214 nm.

Interestingly, we measured a considerable reduction in the adhesion between nanofibers and biomimetic foils between ~ −35% and ~ −62% over the whole test range of spatial periods from ~203 nm to ~613 nm. Though the significantly higher ripples on PET performed somewhat better than the shorter ones on SU-8, this difference is not statistically significant. Ripple height on SU-8 could most probably be increased by using more laser pulses. Theoretical results indicate that, at a given spatial period, higher structures are advantageous because of the more favorable aspect ratio (Joel et al., 2020; Lifka et al., 2022). Joel et al. (2020) modeled the interaction between nanofibers and nanoripples on the calamistrum using an energy-based approach that considers the potential energy of the deflected fiber according to the Bernoulli-Euler beam theory *U*_B_, the potential energy of a fiber stressed by a longitudinal force *S* according to the theory of strings *U*_S_, and the interaction energy due to van der Waals forces *U*_vdW_. The contributions to the potential energy *U* = *U*_B_ + *U*_S_ render the deflected state unfavorable because they increase the total energy. In contrast, the van der Waals energy has a negative sign, which makes the deflected state of the fiber and its attraction to the surface favorable. It is therefore important to determine whether it is the potential energy or the van der Waals energy that dominates in the system under consideration. To quantify whether a surface is adhesive or antiadhesive, we calculate the ratio of the potential energy to the negative van der Waals energy by

$$\text{Γ}=\frac{U}{-U_{\text{vdW}}}=\frac{\frac{h^{2}}{4}\pi^{4}(\frac{4EJ}{{\text{Λ}}^{3}}+\frac{S}{\text{Λ}})}{\sqrt{2R}\frac{A_{\text{H}}}{24d^{1.5}}\max(\text{Λ},2h)},$$

where *Γ* > 1 means antiadhesion and *Γ* < 1 adhesion. In this formula, *E* = 80 MPa denotes the Young’s modulus of a fiber, *J* = *π* × *R*^4^/4 the second moment of area of a fiber with a radius *R*, *A*_H_ = 45 × 10^−21^ J the Hamaker constant and *d* = 0.165 nm the distance at which van der Waals forces become relevant. Considering the spatial periods and heights of the nanoripples (Tables 1, 3), we find that without a preload on the fiber (i.e., with *S* = 0), the smaller fibers with *R* = 7.5 nm adhere to all surfaces, while the larger ones with *R* = 15 nm do not, with the exception of ripples with a spatial period of 613 nm. The fibers of *U. plumipes* have a mean radius of *R* = 10 nm (Joel et al., 2023), which would make about half of the structures adhesive – mainly those with smaller spatial periods. For a preload of *S* = 1 nN, none of the fibers adhere to any of the tested surfaces. According to this theory, the dimensions of the fabricated structures are around the transition point between adhesion and antiadhesion if no load is applied. A longitudinal force, - that is, a preload of the fibers in the natural model – can be assumed in the case of the calamistrum (Lifka et al., 2023), although it has not yet been measured. It has to be emphasized that the exact material parameters are unknown and that the values listed above are estimates. This means that only the qualitative behavior is captured. In addition, it has been shown that the adhesion of these cribellate nanofibers to prey is based not only on van der Waals forces but also on interaction with epicuticular waxes on the prey surfaces (Bott et al., 2017).

Even irregular ripples fabricated without a polarizer reduced the measured maximum deflections before detachment by about ~ −58%. The measurement results indicate that the utilized biomimetic antiadhesive effect is robust against variations in the spatial periods. For future applications, this means that an antiadhesive effect can be achieved despite deviations caused by errors or limitations in production processes, which simplifies manufacturing. As both base materials tested, SU-8 and PET, are widely used technical polymers, we envision numerous applications in tools for example, throughout nanofiber handling. It has to be considered that in this work electrostatic attraction was minimized by gold layers on the nanorippled foils and by the use of ion guns, if necessary. This must be taken into account in future practical applications.

Nanoripples with periodicities matching the range of the LIPSS produced were found on various body parts, namely the calamistra, cuticles, tarsal claws, and spinnerets, of all three cribellate spider species investigated (*Uloborus plumipes, Amaurobius similis*, and *Menneus superciliosus*). That nanoripples are responsible for the antiadhesion between calamistrum and nanofibers has already been demonstrated in previous studies (Joel et al., 2020; Meyer et al., 2021; Lifka et al., 2022). That this antiadhesive effect persists with structural variations in artificial samples suggests that ripple-mediated antiadhesion is not limited to the calamistrum, but is likely also to be found elsewhere. Body parts such as the tarsal claws and the spinnerets of cribellate spiders frequently come into contact with nanofibers, be it during production, maintenance of the web, or interaction with prey or predators (Peters, 1984; Eberhard, 2020). Therefore, an antiadhesive character of these structures is highly plausible. In contrast to the cribellate spiders investigated, which catch their prey with adhesive bundles of nanofibers, ecribellate spiders use glue droplets instead for this purpose (Vollrath, 2000). Analogously, it has been reported that their body has an antiadhesive coating to prevent them from adhering to their own threads (Kropf et al., 2012).

Similar nanorippled structures have been found on the bodies of other cribellate species (Platnick et al., 1991; Griswold et al., 2005; Ramírez, 2014; Labarque et al., 2017). We assume these antiadhesive structures on exposed body parts to be a universal feature of most cribellate species. Future studies should look deeper into the occurrence as well as the dimensions of the nanoripples and measure their antiadhesive properties directly. As a suitable negative control for our hypothesis, the previously mentioned ecribellate spiders could be used, i.e., those that do not use nanofibers but glue. They should not exhibit nanoripple-mediated antiadhesion.

Considering that less regular structures presumably achieve equivalent effectiveness, the question arises as to the necessity of the delicate and conserved nanotopography of the calamistrum. It may be linked to the intense contact between the calamistrum and freshly synthesized nanofibers or to other, as yet unknown processing features of the calamistrum. Cribellate spiders may also use excessive grooming to compensate for inferior antiadhesion of body parts with less accurate nanoripples. We observed that the two species *U. plumipes* and *Kukulcania hibernalis* clean their tarsi and spinning apparatus – but not the calamistrum – of fiber residues during web production (unpublished observation).

## 5 Conclusion

Antiadhesion between the calamistrum and nanofibers with diameters between 15 nm and 30 nm is thought to be due to a special fingerprint-like nanotopography of the comb. We found similar structures, but with different spatial periods, on many other body parts of cribellate spiders and assessed their dimensions. Varying the spatial periods of biomimetic nanoripples on PET and SU-8 by changing the angle of incidence of the laser beam during processing did not compromise the antiadhesive properties of the resulting foils. We have thus demonstrated that this effect is robust against variations of the spatial periods within a certain range. With one exception, the investigated range comprises the distances of the ripples found on the spiders´ various body parts. Characterization of this behavior will facilitate the use of such biomimetic antiadhesive surfaces in technical applications, as it will reduce the requirements of the setup for surface structuring. Since SU-8 and PET are widely used in research and industry, possible application areas are abundant. In the case of SU-8, laser-processing (i.e., LIPSS) and standard UV photolithography can even be combined. Artificial antiadhesive surfaces may be applied in tools for handling nanofibers such as spoolers for single nanofibers, conveyor belts for producing endless nanofiber nonwoven, and cylindrical tools for fabricating tubular nanofiber nonwoven or endless nanofiber nonwoven (Lifka et al., 2023). Applications of this approach in the field of alternative fibers such as carbon nanotubes (Shariatinia, 2021) remain to be explored.

## Figures and Tables

**Table 1:** Dimensions of biomimetic nanoripples on PET and SU-8 covered with a thin gold layer at one exemplary position at the sample: mean spatial periods *Λ* ± standard deviations depending on the various angles of incidence *θ* of the laser beam; for irregular ripples, only a mean peak-peak distance *d*_P-P_ and its standard deviation were calculated.

Angle of incidence ***θ*** (°)	0	0 (irregular)	30	60
PET				
Spatial periods *Λ* (nm) or peak-peak distance *d*_P-P_ (nm)	214.1 ± 0.6 (±0.3%)	274 ± 91 (±33%)	330.7 ± 0.2 (±0.2%)	613 ± 5 (±0.8%)
SU-8				
Spatial periods *Λ* (nm)	202.9 ± 0.6 (±0.3%)	-	330 ± 1 (±0.3%)	-

**Table 2:** Dimensions of biomimetic nanoripples on PET and SU-8 covered with a thin gold layer at five exemplary positions at one sample: mean spatial periods *Λ* ± standard deviations of biomimetic nanoripples on PET and SU-8 fabricated with an angle of incidence *θ*=30°.

Measurement position	1	2	3	4	5	Mean of # 1–5
PET (*θ* = 30°)						
Spatial periods *Λ* (nm)	325.3 ± 0.3 (±0.1%)	326.2 ± 0.1 (±0.04%)	330.7 ± 0.2 (±0.07%)	319.2 ± 0.4 (±0.1%)	334.2 ± 0.1 (±0.04%)	327 ± 6 (±2%)
SU-8 (*θ* = 30°)						
Spatial periods *Λ* (nm)	299 ± 2 (±0.7%)	327 ± 2 (±0.5%)	330 ± 1 (±0.3%)	313 ± 1 (±0.4%)	319 ± 2 (±0.5%)	318 ± 12 (±4%)

**Table 3:** Heights *h* ± standard deviations of biomimetic nanoripples on PET and SU-8 covered with a thin gold layer at two exemplary positions at the sample: mean spatial periods *Λ* ± standard deviations of biomimetic nanoripples on PET and SU-8 depending on the various angles of incidence *θ* of the laser beam; evaluation of 20 ripples at each position.

Angle of incidence ***θ*** (°)	0	0 (irregular)	30	60
PET				
Heights *h* at position 1 (nm)	97 ± 46 (±47%)	110 ± 36 (±33%)	161 ± 21 (±13%)	122 ± 41 (±34%)
Heights *h* at position 2 (nm)	63 ± 21 (±33%)	118 ± 39 (±33%)	112 ± 18 (±16%)	150 ± 48 (±32%)
SU-8				
Heights *h* at position 1 (nm)	63 ± 8.8 (±14%)	-	73 ± 7.1 (±10%)	-
Heights *h* at position 2 (nm)	69 ± 5.2 (±7.4%)	-	75 ± 14 (±18%)	-

**Table 4:** Morphology of nanoripples found on different body parts in three spider species.

	Calamistrum	Cuticle	Tarsal claw	Spinneret
*U. plumipes*	200 ± 35	751 ± 69	462 ± 90	552 ± 89
*A. similis*	224 ± 27	287 ± 35	623 ± 81	511 ± 92
*M. superciliosus*	198 ± 40	374 ± 51	326 ± 68	631 ± 59

Per species, 15 measurements were taken in up to three specimens. Listed are the mean spatial periods *Λ* ± standard deviations of naturally occurring nanoripples found on different body parts of *U. plumipes, A. similis*, and *M. superciliosus*. All values are given in nm.

**Table 5:** Indirect measurement of antiadhesion (by maximal deflection of nanofibers) of gold-covered nanorippled SU-8 and PET surfaces, presented as mean values ± standard deviations and relative reductions of the mean values compared to the reference samples fabricated from the very same materials.

Sample type	Deflection (μm)	*p*-value	Relative reduction (%)
SU-8: 0°/203 nm	458 ± 180	1.81e-05	−43
PET: 0°/214 nm	434 ± 177	3.98e-06	−54
SU-8: 30°/330 nm	520 ± 253	9.20e-04	−35
PET: 30°/331 nm	432 ± 160	3.50e-06	−54
PET: 60°/613 nm	360 ± 176	1.20e-06	−62
PET: 0° (n.p.)/irregular	397 ± 202	3.50e-06	−58

For each sample type, 20 measurements were conducted. The reference values are, respectively, 801 μm ± 194 μm for SU-8 and 937 μm ± 299 μm for PET. LIPSS and control data were compared using a Wilcoxon rank sum test; the resulting *p*-values are listed, and significance was assumed if *p* < 0.05.

**Table 6:** Determination, of whether the deflection measurement data can be assumed to be normally distributed.

*p*-value	Kolmogorov–Smirnov test	Lilliefors test
SU-8: 0°/203 nm	0.8934	0.5000 (greater than the largest tabulated value)
PET: 0°/214 nm	0.8202	0.4309
SU-8: 30°/330 nm	0.7284	0.2988
PET: 30°/331 nm	0.4485	0.0693
PET: 60°/613 nm	0.4728	0.0811
PET: 0° (n.p.)/irregular	0.4903	0.0904
PET: reference	0.7324	0.3035
SU-8: reference	0.5547	0.1324

The given *p*-values were assessed by Kolmogorov–Smirnov and Lilliefors distribution tests and significance was assumed if *p* < 0.05.

**Table 7:** Matrix with *p*-values from multiple pairwise comparisons of the mean values from the indirect measurement of adhesion (by deflection) using Tukey’s honestly significant difference criterion; the default significance level of *p*<0.05 was used.

*p*-value	SU-8: 0°/203 nm	PET: 0°/214 nm	SU-8: 30°/330 nm	PET: 30°/331 nm	PET: 60°/613 nm	PET: 0° (n.p.)/ irregular	PET: reference	SU-8: reference
SU-8: 0°/203 nm	-	1.0000	0.9835		0.8176	0.9835	5.9893e-08	7.3626e-06
PET: 0°/214 nm	1.0000	-	0.8991	1.0000	0.9552	0.9993	5.9882e-08	9.9333e-07
SU-8: 30°/330 nm	0.9835	0.8991	-	0.8925	0.2369	0.5828	6.8832e-08	6.4464e-04
PET: 30°/331 nm	0.9999	1.0000	0.8925	-	0.9589	0.9995	5.9882e-08	9.0539e-07
PET: 60°/613 nm	0.8176	0.9552	0.2369	0.9589	-	0.9993	5.9881e-08	6.0723e-08
PET: 0° (n.p.)/ irregular	0.9835	0.9993	0.5828	0.9995	0.9993	-	5.9881e-08	9.3384e-08
PET: reference	5.9893e-08	5.9882e-08	6.8832e-08	5.9882e-08	5.9881e-08	5.9881e-08	-	0.4418
SU-8: reference	7.3626e-06	9.9333e-07	6.4464e-04	9.0539e-07	6.0723e-08	9.3384e-08	0.4418	-

## Data Availability

The raw data supporting the conclusions of this article will be made available by the authors, without undue reservation.

## Abbreviations

2D: two-dimensional
ANOVA: analysis of variance
FE-SEM: fieldemission scanning electron microscope
FFT: fast Fourier transform
FIB: focused ion beam
HDMS: hexamethyldisilazane
LIPSS: laser-induced periodic surface structures
ns: nanosecond
PET: poly(ethylene terephthalate)
rpm: rotations per minute
SEM: scanning electron microscopy
UV: ultraviolet
